# Teaching, coaching, or debriefing With Good Judgment: a roadmap for implementing “With Good Judgment” across the SimZones

**DOI:** 10.1186/s41077-022-00235-y

**Published:** 2022-11-26

**Authors:** Mary K. Fey, Christopher J. Roussin, Jenny W. Rudolph, Kate J. Morse, Janice C. Palaganas, Demian Szyld

**Affiliations:** 1 George Washington University, Washington, D.C USA; 2 Center for Medical Simulation, Boston, MA USA; 3 Department of Anesthesia, Critical Care, & Pain Medicine, Massachusetts General Hospital, Boston, MA USA; 4 Harvard Medical School, Boston, MA USA; 5 School of Nursing, Drexel University, Philadelphia, PA USA; 6 MGH Institute of Health Professions, Boston, MA USA; 7 Department of Emergency Medicine, Boston University School of Medicine, Boston, MA USA

**Keywords:** SimZones, Healthcare simulation, Teaching and learning strategies, Learning conversations, Faculty development, Health professions education, Debriefing

## Abstract

Simulation-based learning occurs in multiple contexts, and one teaching style cannot adequately cover the needs at each learning level. For example, reflective debriefing, often used following a complex simulation case, is not what is needed when learning new skills. When to use which facilitation style is a question that educators often overlook or struggle to determine. SimZones is a framework used to clarify the multiple contexts in simulation. This framework, combined with elements of Debriefing With Good Judgment, can help educators match the appropriate facilitation style with learner needs and learning context. We have distilled the core elements of the “with good judgment” approach to debriefing and applied them to the SimZones framework to guide educators with (1) what type of learning can be expected with each learning context, (2) what behaviors and activities can be expected of the learners in each learning context, (3) what instructional strategies are most effectively used at each stage, and (4) what are the implications for the teacher-learner relationship.

## Manuscript

Well-intentioned simulation educators create ineffective learning experiences when curriculum design, learners’ needs, and the facilitation method do not align. Ambiguity about the teacher and learner roles can rob both of self-confidence, can leave both feeling disappointed and angry, and erode their relationship. The simulation educator is often uncertain when to use directive teaching versus facilitated reflection. Mismatches between learners’ expectations and needs and the instructional approaches can result in missed opportunities for learning, erosion of instructor confidence, or worse. Consider these mismatches as follows:

- A learner is struggling with the desirable difficulties [1] of mastering a new skill. They need clear direction to take the next step. Instead, the well-intentioned instructor asks open-ended, pseudo-Socratic questions that leave the student wondering what the instructor wants them to say or do [2].
- A group of experienced learners working on the challenges inherent in a complex teamwork scenario would benefit from guided reflection to help them examine assumptions and cognitive biases that impeded their teamwork. Instead, the instructor “teaches” the ACLS algorithms they already know, yielding no new learning, and leaving learners with a feeling of wasted time.

The educator may err on the side of too much “telling” when critical self-reflection is needed, or, conversely, may not provide enough direct instruction as learners work to master new skills [3].

Simulation-based educators face many decision points. They must know when to employ which instructional strategy, and it can be unclear what to do. When should I just teach and tell? How do I help as expertise in developing yet some guidance is still needed? When should I take a position of facilitating reflection and co-creating learning experiences? Recognizing the person leading the learning experience will need to assume a different stance based on the learning context is often not made explicit and may not be intuitive to the less experienced educator. Telling when they should be facilitating or facilitating when they should be telling are both ineffective, and both can result in frustrated learners and instructors. Both sides may experience violations of their expectations, which can degrade the teacher-learner relationship [4].

Health professions educators apply simulation-based learning in diverse learning situations. It is unrealistic to think that curriculum and instructional strategies would be the same across all contexts. Simulation-based learning suits multiple purposes across the developmental trajectory of a clinician: learning foundations skills, practicing and mastering those skills in realistic contexts, and continually developing individual and team-based skills [5]. Matching curricular and instructional approaches with precision is a potent way to meet learners’ needs.

We will use the term “learning leader” to capture the diverse roles played by the simulation-based instructor across different levels of learning. We use this term instead of instructor for several reasons. First, we recognize that simulation faculty engage in a range of activities, including directive teaching, individual coaching, and facilitating self-reflection. Second, the Learning Leader is responsible for more than just the curricular content; they lead the entire experience, such as pre-simulation preparatory work, creating and maintaining psychological safety, the pre-simulation briefing, the implementation of the simulation activity, and the post-simulation learning conversation. Third, learning leaders take on the responsibilities of leaders in other realms, such as in business or organizational contexts in that they influence the activities of an organized group toward goal achievement [6]. For example, the Learning Leader can affect the group’s activities by creating a learning environment rich in psychological safety so that experiential learning can thrive [7–9] and customize teaching activities to suit the learning and moment [5].

Novice learners need more support and direction from the learning leader; as expertise develops, a less directive stance allows the learner to more fully co-create the experience with the Learning Leader [10]. We did not locate any literature related to human patient simulation that provides guidance to direct learning leaders on what stance (teacher, coach, or facilitator) they should take as they relate to learners across the span of expertise and simulation types.

To better align learning needs with instructional strategies, we integrate the SimZones curricular approach [5] with the essential elements of the Debriefing With Good Judgment method [11] to provide a roadmap for learning leaders. This article delineates the elements of “With Good Judgment,” describes the learning pathway along which learners progress using the SimZones framework, and describes three distinct approaches: *teaching with good judgment, coaching with good judgment, and debriefing with good judgment*.

Using the lens of social constructivist learning [12, 13], which describes a dynamic and developmental relationship between learner and context, we see SimZones as the staged contexts in which teaching and learning are experienced. Within the zones, the “With Good Judgment” approach provides a developmental context via its nurturing emphasis on curiosity and respect for the learner as well as clarity about the standards to be met. This article aims to clarify expectations and goals for the Learning Leader and offers guidance by answering the following questions:

1. What is the stance of the Learning Leader in each SimZone?
2. What type of learning should I expect in each SimZone?
3. What behaviors and activities should I expect of the learners in each SimZone?
4. What instructional strategies should the Learning Leader use in each SimZone?

## Background

Learning is a social process [12–16]. Cognitive development stems from the social interaction between educator and learner throughout the learning process [14]. In this complex and nuanced relationship, the Learning Leader is responsible for crucial aspects of learning: preparing the learning context, orienting the participants, leading the simulation activities, and supporting self-reflection through learning conversations. A phenomenon that exemplifies these relationships is the “zone of proximal development” in which the Learning Leader scaffolds the learner in a way that allows the learner to take steps they might not have taken on the their own [14, 16].

Health professions education now recognizes debriefing as the reflective learning component of simulation-based education. Debriefing synthesizes learning, creates the opportunity for the learner to engage in critical self-reflection, provides a means to give feedback, and explores individual and team cognition [17–23]. However, simulation educators often find that debriefing is not what learners need in certain learning situations, such as when teaching new skills or when helping individuals hone newly acquired but decontextualized skills in the clinical learning lab [5]. Recognizing the learning context and knowing what to do in that context are critical for achieving learning outcomes throughout the simulation-based education continuum.

### Elements of the With Good Judgment approach

Being learner centered is often a goal of educators [24, 25]. Learner-centered teaching is an approach in which control of the learning experience shifts away from the teacher to the learner. Approaches such as active learning and problem-based learning are examples of this. These approaches have been shown to increase student motivation and retention of knowledge [24, 25]. A sincere desire to be learner centered can inadvertently result in ineffective learning leader behaviors. Wanting to avoid being “the sage on the stage” [26], they may be reluctant to “just tell them how to do it” and instead try to gently steer learners to the correct action, “hinting and hoping” that learner will figure it out. This approach can be frustrating to both the learner, who just needs some help figuring out how to perform a skill and the learning leaders who do not feel free to share their expert judgment for fear of being perceived as harsh or “judgmental.” Learning leaders can resolve the internal dilemma by applying the core elements of the With Good Judgment approach.

In the 15 years since the publication of the seminal article [11], our team of diverse educators has distilled the core elements of the “With Good Judgment” approach. The core elements are as follows: (1) holding learners to high standards while holding them in high regard; (2) transparency in communication on the part of the instructor, by narrating your thinking (i.e., sharing your goals, saying what you see, describing what you think); and (3) conversational strategies that match the intended learning outcomes.

### High standards, high regard

The first element, holding learners to high standards while holding them in high regard, is an internal shift, on the part of the educator, in how they view the learner. Often, if a learner falls short of the standard, learning leaders condemn the lapse, treating it as a crime to be punished. To hold the learner in high regard is to generously view them as intelligent, capable individuals who are sincerely trying to do their best and want to get better. This high regard assumes the learner can meet the standard with the right support. Education theorists and psychologists have long embraced the idea that this type of positive regard toward and empathy for the learner is essential to constructivist active learning environments [9, 27, 28]. Believing that learners are sincerely trying to do their best and want to get better is a powerful belief that, once genuinely incorporated into the educator’s teaching philosophy, transforms learning conversations. For the learning leader, holding the learners in high regard defuses the “bomb” of negative judgment. If the Learning Leader genuinely assumes the best of the learner, it frees them from the exhausting work of hiding any negative feelings. Even temporarily assuming the best of the learner allows the Learning Leader to take a curious and respectful stance toward the learner, which in turn releases them to share their expert judgment, even when critical, to encourage self-reflection on the part of the learner. When learners experience this high regard and genuine curiosity consistently, it reduces their fear that mistakes and struggles will be met with shaming or humiliation. The Center for Medical Simulation’s Basic Assumption [29] captures the simultaneous ideas of a high standards and high regard. It states the following: “We believe that everyone participating here today is intelligent, capable, cares about doing their best, and wants to improve.”

### Transparent thinking

The second element of the With Good Judgment approach is “transparent” thinking, the idea that it is valuable to narrate what you think. With this approach, Learning Leaders share their thoughts explicitly. They preview the topic of focus, share concrete observations about the learners’ performance, and explicitly share their take on the clinical implications of the performance. These attributes of the With Good Judgment approach lay the foundation for clear feedback [30]. Being transparent about the topic at hand allows the learner to focus their attention on a specific aspect of the situation or performance. As the Learning Leader advocates for their own point of view, they describe their view of what they observed and the impact of the performance [11]. For example, imagine a simulation scenario where the learner encounters a simulated patient who informs them that do not want to adhere to the pharmacologic pain management protocol in favor of “natural” remedies. During the encounter, the learner bluntly tells the patient that those remedies will not work. The Learning Leader may open the conversation by saying the following:

- Preview: “I’d like to discuss the value of including the patient in the plan of care.”
- Instructor’s observations (“advocacy”): “Nathan, I heard you tell the patient, Well, 15mg of morphine is the right dose for you. You should take it. Those herbs and teas are not going to relieve your pain.”
- Instructor’s concerns (“advocacy”): “I’m thinking that she will not feel her concerns were heard and she’ll feel excluded from her own care decisions. This could lessen the chance that she’ll adhere to the plan.”

#### Varying conversational strategies

The third element of the With Good Judgment approach is varying conversational strategies that match the expected outcome. For example, when skill acquisition is the expected outcome, the instructional approach of directive teaching is used. Conversely, when reflection on thought processes and team interaction is the expected outcome, a debriefing approach is used. These conversational strategies are discussed below and are mapped to SimZones in Table 1.

**Table 1:** Applying with good judgment across the SimZones

		Simulation zone		
		**Zone 1: procedure and skills workshops**	**Zone 2: acute situational instruction**	**Zone 3: team and system development**
**Learning leader’s stance**	**Goal of experience**	Learn basic domain-specific skills	Practice skills in context	Continuous development of individuals and teams
	**What learning happens**	Acquiring and demonstrating skills such as the following: • Clinical • Conversational • Teaming Patient assessment	How to recognize and manage situations using zone 1 skills in context such as the following: • Anesthesia induction in an obese patient • Escalating care using “SBAR”	Clarifying the underlying barriers to and corrective actions for high performance using the unique perspectives of participants such as the following: • Managing a failed airway • Team-based management of massive hemorrhage
	**Learning domain**	Skill acquisition: e.g., psychomotor, conversational, teaming, patient assessment	Skills are refined. Performance of a skill with contextual demands and higher cognitive load	Co-creation of learning and change by identifying personal and team strategies
	**How to create and sustain the trusting learning environment?**	I create trust by sharing expertise but also model that I am also still learning and refining my own practice. I have many but not all the answers. I signal openness humility to remain approachable despite my expertise	I create trust by being dependable and honest and holding a high standard and sharing it with compassion. I use predictable direct feedback techniques. I avoid shaming or showing up the learner	I create trust by giving up control and authority and position myself as an external helper, observer and supporter of the team, and offer insights if and when invited
		**Zone 1: procedure and skills workshops**	**Zone 2: acute situational instruction**	**Zone 3: team and system development**
**In-zone activity**	**Learning conversation strategies**	**Teaching with good judgment:** preview, advocacy, teach Modeling and demonstration and asking guiding questions and sharing direct feedback Identify developmental next step and make prescriptions for future practice	**Coaching with good judgment:** *preview*, *advocacy*, *coach* Providing expert feedback, encouraging successes, and motivating high-performance standards Propose discussion agenda and then inquire, listen, and coach to needs	**Debriefing with good judgment:** *preview*, *advocacy*, *inquire* Coordinating reflective conversations and fostering dialogue among team members Use inquiry to explore mental models, assumptions, and biases that drove participant’s actions
	**Role of the learning leader**	The expert — holder of knowledge and a master of the standard. Controls the experience to a great extent	A colleague with valuable expertise. Guides many elements of experience and may negotiate some such as topic selection or time allocation during reflection	A facilitator of reflection. Helps team recording findings and synthetizing next steps. Coordinates the conversation and shares their expertise sparingly
	**Role of the learner**	Seeker of knowledge & skill	Beginning practitioner	Competent practitioner(s) striving for continuous improvement

### Learning contexts: SimZones

SimZones, introduced by Roussin and Weinstock [5], provide an instructional framework to map and facilitate the learning progression from novice to competent practitioner. They describe simulation zones 0–4, with learning goals and facilitator approaches in each. Zone 0 includes facilitator-free instruction with automated devices or computerized programs the give feedback. Zone 1 is the context in which foundational skills are taught whether they be psychomotor, communication, or teaming skills. In zone 2, learners practice recently acquired skills in important situational contexts. Zone 3 involves simulation for the ongoing development of individuals, teams, and systems. Zone 4 is real-world practice. Because this manuscript targets simulation-based education in which an instructor is present, our discussion will focus on zones 1, 2, and 3. The following section integrates the with good judgment approach with the SimZones by describing the Learning Leader stance and key skills for each SimZone.

### Zone 1: Teaching With Good Judgment

Teaching With Good Judgment is combined with widely accepted approaches to learning skills such as deliberate practice [31–33], mastery learning [34, 35], or rapid cycle deliberate practice [36, 37]. With little or no context for the skills, participants’ primary need is clear direction. The Learning Leader’s decisions are guided by the pursuit of learning outcomes identified for each activity in the curriculum.

#### Learning Leader stance

The Learning Leader is a respectful and curious expert, here to introduce learners to current best practices. The hierarchy is seen as positive. To hold the learner to high standards while holding them in high regard, the Learning Leader believes that the learner can meet the standard and sees the learner as intelligent, capable, and wanting to improve. Mistakes are treated as inevitable and welcome steps in the learning process. The Learning Leader sees feedback as developmental (rather than critical) and recognizes that a psychologically safe learner is more capable and motivated [38].

#### Learner preparation

The Learning Leader is transparent about the session goals and learner responsibilities by previewing them clearly. They prepare learners for success by reviewing the criteria by which success will be determined. They explicitly discuss logistics, objective, and performance goals and help the learners understand how to “act as if” the simulation is real while acknowledging that it is not perfect (i.e., the fiction contract [39]) during a briefing before the simulation experience.

#### In-zone activities

During the skills workshop, Learning Leaders model the skill, preview what to expect (e.g., what aspects will be challenging, what common pitfalls are, and how to avoid them), and invite questions. They normalize by relating their own experience with learning the skill including their struggles. They observe practice and identify points of performance where help is needed. They share expert judgment through direct teaching.

Imagine a simulation-based learning experience in which intubation is being taught and the learner’s technique risks breaking the patient’s teeth as follows:

- Preview the topic: “Let’s talk about handling the laryngoscope in a way that protects the patient’s teeth.”
- Instructor’s observation(s) (advocacy): “I see that you’re pushing against the patient’s teeth as you’re trying to intubate.”
- Instructor’s concern(s) (advocacy): “In a real patient, this downward pressure might chip a tooth.”
- Adaptive conversational strategy — teach: “Pull in an upward direction, avoiding pressing against the teeth. Instead of rocking the handle back, pull up and away from you to open the airway.”

The zone 1 conversational strategy is preview-advocacy-teach

#### Post-simulation

Learning Leaders can end the session with a brief reflection. Inquire about challenges encountered and help identify and recommend specific practice for the next developmental step.

### Zone 2: Coaching With Good Judgment

#### Learning Leader stance

The Learning Leader is a respectful and curious expert, here to coach learners up the learning curve to achieve current best practice. Learners come to the session with baseline skills and require individualized coaching to achieve the preset standard as they apply new skills in various realistic contexts. They will now practice those skills in an immersive realistic context, adapting those skills to the demand s of the situation. For example, the learner who is advancing their intubation skills will now encounter a challenging airway in a scenario that mimics the clinical environments. Coaching and reflection can occur by pausing the simulation periodically to conduct a micro-debriefing [40] or during a post-simulation coaching and debriefing conversation. The individual’s performance guides the Learning Leader’s customized decisions. As in athletic coaching, the Learning Leader must observe for flaws and strengths in practice and provide corrective and supportive feedback to improve or sustain performance [41]. Landreville et al. [42] describe the transfer of these principles from athletics to developing clinical skills in medical education. Similar to coaching an athlete, coaching involves observation with specific feedback and actionable suggestions for performance improvement.

Suppose a learner is struggling with a core skill in this new context. The Learning Leader should determine if the challenge lies in executing the skill itself or if the added cognitive load from attending to the other elements in the clinical situation is causing the problem. In the former case, the learner may need further Zone 1 teaching. If the latter, the Learning Leader may provide corrective guidance about executing the skill despite the distractions and additional cognitive load the realistic context brings. Progression through the zones may not be linear for everyone. Grappling with the desirable difficulties at any level helps people learn and retain new skills. This is expected and is not a failure [1].

#### Preparation of learners

Learners are prepared for success as the Learning Leader connects the skills acquired in Zone 1 to the learning activities and outcomes of the current Zone 2 simulation experience. Transparency in learning objectives and how the simulation will be executed (e.g., there may be planned pauses during the simulation, and that struggle and awkwardness are normal) will maintain psychological safety for the participants.

#### In zone activities

The Learning Leader observes and notes challenges, successes, and errors. The Learning Leader may pause the simulation to provide customized coaching or prepare the learners for the next steps. They may also allow the simulation to run without interruption and provide only post-simulation coaching. Coaching involves suggesting strategies tailored to the individual’s challenges as they now perform to the standard in the new environment. They incorporate the principles of high standards and high regard, as well as transparency on the part of the educator. While the preview and advocacy parts of the learning conversation remain the same as in Zone 1, those strategies are now combined with coaching. For example, imagine that a Zone 2 training asks the learners to care for a patient experiencing respiratory failure who needs emergent intubation. The learner attempts intubation with the bed in the low position and is unable to intubate successfully.

- Preview the topic: “Let’s discuss the impact of bed position on intubation.”
- Instructor’s observation(s) (advocacy): “I see that you’re attempting to intubate with the bed in the low position.”
- Instructor’s concern(s) (advocacy): “I think that may be why you’re having a hard time visualizing the cords. It also puts you at risk for a back injury.”
- Adaptive conversational strategy — coach: “Let’s start again, and this time, get the patient and yourself in a better position before you attempt the intubation.”

**The Zone 2 conversational strategy is preview-advocacy-coach**

Post-simulation: The Learning Leader may use this approach to coaching in a post-simulation learning conversation or during a pause in the simulation. Post-simulation, the Learning Leader sets the discussion agenda with the learners by proposing topics to discuss. There may be opportunities to inquire, listen, and coach by sharing expertise, generating discussion, and sharing insights. Provide opportunities to try again and help learners identify strategies to improve future performance.

### Zone 3: Debriefing With Good Judgment

#### Learning Leader stance

The Learning Leader is a respectful and curious expert facilitator (with or without subject matter expertise). They are here as a caretaker and conversation guide to promote reflection on and discussion about the barriers and facilitators to reaching the best outcomes. Learners are autonomous actors and bring a strong baseline of skills and experience to the simulation. They are meaning makers, not simply doers of correct or incorrect actions. In a hospital context, this is about continuous improvement of teams and systems. In pre-licensure educational settings, this might take the form of reflective conversations designed to strengthen critical thinking or to prepare for transition into practice, as students have acquired one level of skill and are moving on to the next. Participants are at the center and drive the experience by being invited to collaborate in setting the priorities for the reflective learning conversation. The Learning Leader is central to the reflective process but does not unilaterally direct the conversation. The Learning Leader’s role is to help the team identify opportunities for improvement, uncover and describe assumptions, evaluate their systems and practices, and grapple with real-world challenges.

#### Preparing the learners

In pre-simulation briefing, create shared ground rules for working together. Of particular importance is a discussion of roles during the debriefing, i.e., the Learning Leader is a facilitator, but the learner’s agenda is considered equal to the Learning Leader’s intended objectives. In particular, the goals and agenda of the debriefing are to explore the aspects of the simulation that the learners found most challenging or the elements of the simulation in which the learners performed exceptionally well.

#### In-zone activities

The Learning Leader observes with an eye toward continuous improvement, noting specific challenges and examples of stellar performance. Also considered are institutional priorities, academic competencies, and team interactions.

#### Post-simulation

During the debriefing, the Learning Leader facilitates a reflective discussion using inquiry questions to explore mental models, assumptions, and biases that drove clinical decision-making. The Learning Leader and the team agree on an agenda, organize the conversation, invite diverse perspectives, and help tolerate and explore ambiguity to help learners achieve or sustain good future performance [39]. As needed, offer possible next steps or alternative strategies. Debriefing would incorporate the principles of high standards and high regard, as well as transparency in thinking on the part of the educator. Combine this educational stance with question aimed at helping learners reflect on their decision-making by understanding the thinking behind the decisions. For example as follows:

- Preview the topic: “Let’s talk about the issues involved in a decision to intubate.”
- Instructor’s observation(s) (advocacy): “I heard you call for intubation meds just moments after you examined the patient.”
- Instructor’s concern(s) or positive feedback: “I think this rapid action avoided a prolonged period of hypoxia, which will ultimately help improve the outcome.”
- Adaptive conversational strategy — Inquire (i.e. about learners’ thought processes): “Can you walk me through your thinking at that moment?”

The Zone 3 conversational strategy is preview-advocacy-inquiry.

## Conclusions

This adaptive approach to simulation-based education integrates the With Good Judgment approach with the curricular strategy of the SimZones. It incorporates the following: the philosophical foundation of holding the learner to high standards while holding them in high regard, the practice of the Learning Leader being transparent in their thinking and communication, and the method of adapting conversational strategies to optimize the learning across the developmental pathway of a clinical learner. Adaptively adjusting when we teach, coach, and inquire to meet the learner where they are reduces troublesome ambiguity from the teacher-learner relationship. Instead of worrying “Am I telling or facilitating?”, “Is this learner-centered or teacher-centered?”, and “Am I the sage on the stage or the guide on the side?”, integrating With Good Judgment across SimZones makes explicit the often-implicit psychological contract between teacher and learner. Knowing what is expected and owed to each other in each zone, the Learning Leader may confidently provide the type and amount of guidance dictated by the leaner’s developmental stage. It provides the learner with the appropriate amount of scaffolding to support early learning and allows for increased autonomy and reflection as expertise develops.

Matching Learning Leader strategies to the expected outcome supports and nourishes the teacher-learner relationship. It allows the Learning Leader and learner to develop a quick and shared mental model of the learning encounter during the pre-simulation briefing. This learning contract reduces the awkwardness of episodes such as when learners expect reflective facilitation but are instead schooled on a subject they think they know or instead are counting on being taught and instead are asked to share their thinking.

We hope this roadmap helps educators and organizations deliberately design and implement simulation-based curricula. Many health systems across the world currently face a crushing workforce shortage. Having a clear, potent roadmap to guide readiness as clinicians develop new skills could be transformational. Across health professions, outputs of education, such as readiness for practice, are in sharp focus. For example, in some countries, medical students’ experiences are guided by Entrustable Professional Activities [43]; nursing and other health profession schools also adhere to standards set by regulatory bodies. The outcome of education, therefore, is not learning but competent practice. We think the With Good Judgment across the SimZones provides the sort of crisp guidance that could streamline and accelerate readiness in a variety of contexts. The roadmap for With Good Judgment and SimZones keeps the learner at the center of the experience in a way that promotes competency and readiness, supports productive feedback, and strengthens the teacher-learner relationship.

## Data Availability

Not applicable
